# “I just want to get better”: experiences of children and youth with juvenile idiopathic arthritis in a home-based exercise intervention

**DOI:** 10.1186/s12969-018-0273-6

**Published:** 2018-09-20

**Authors:** Joanie Sims-Gould, Douglas L. Race, Heather Macdonald, Kristin M. Houghton, Ciarán M. Duffy, Lori B. Tucker, Heather A. McKay

**Affiliations:** 1 University of British Columbia, Vancouver, Canada; 2 British Columbia Children’s Hospital, Vancouver, Canada; 3 University of Ottawa, Ottawa, Canada; 4 Children’s Hospital of Eastern Ontario, Ottawa, Canada; 5 Centre for Hip Health and Mobility, 2635 Laurel Street, Vancouver, BC V5Z 1M9 Canada

**Keywords:** Barriers and facilitators, Juvenile idiopathic arthritis, Exercise, Intervention, Qualitative methods

## Abstract

**Background:**

Physical activity is essential for ensuring optimal physical function and fitness in children with juvenile idiopathic arthritis (JIA). Although exercise intervention trials informed current clinical practice, few studies addressed why children with JIA do or do not participate in exercise interventions. We aimed to describe perceived barriers and facilitators to the uptake and adherence to a 6-month home-based exercise intervention for children diagnosed with JIA and their parents.

**Methods:**

A convenience sample of children (*n* = 17) and their parents (*n* = 17) were recruited from a group of 23 child-parent dyads participating in an exercise intervention study; the Linking Exercise, Activity and Pathophysiology Exercise Intervention (*LEAP-EI*) study. Child-parent dyads completed in-depth semi-structured one-to-one interviews with a trained interview moderator prior to starting the exercise program and 11 dyads completed follow-up interviews at the end of the 6-month program. We also conducted ‘exit’ interviews with one child-parent dyad, one child and one parent following three participants’ withdrawal from the exercise intervention. Interviews were transcribed and transcripts were analyzed using a five-step framework analysis to categorize data into themes.

**Results:**

Thematic analysis of pre-exercise program interview transcripts revealed three reasons child-parent dyads initiated the exercise program: 1) *potential health benefits*, 2) *selflessness* and 3) *parental support*. Analysis of post-exercise intervention transcripts identified four main themes within a priori themes of barriers and facilitators to program adherence (median of 46.9%; 5.4, 66.7 IQR): 1) *parental support*, 2) *enjoyment*, 3) *time pressures* (subthemes: time requirement of exercise, scheduling, forgetting) and 4) *physical ailments*.

**Conclusion:**

Major barriers to and facilitators to exercise for children with JIA fell into three categories: personal, social and programmatic factors. These barriers were not unlike those that emerged in previous exercise intervention trials with healthy children and youth. There is a need to develop effective strategies to engage children in physical activity and to overcome barriers that prevent them from doing so. Future initiatives may potentially engage children in developing solutions to enhance their participation in and commitment to physical activity.

## Background

Physical activity and structured exercise^1^ [1] are important components of juvenile idiopathic arthritis (JIA) management [2]. Current research suggests exercise therapy may improve physical fitness, functional ability and quality of life, and reduce pain among children with JIA [3, 4]. In previous intervention trials, children with JIA tolerated prescribed exercise well, and participant withdrawal due to pain was rare. Results of these intervention trials informed current clinical practice for structured exercise in children with JIA, yet very few [5, 6] addressed the important question of why or why not children with JIA actually choose (or not) to participate. This information is necessary to facilitate development of effective exercise programs for children with JIA.

In the *Linking Exercise, Activity and Pathophysiology in juvenile idiopathic arthritis: Exercise Intervention (LEAP-EI) study* we aimed to address this knowledge gap by identifying barriers and facilitators to uptake of, and adherence to, a 6-month home- and group-based exercise intervention for children and youth with JIA.

## Methods

### Participants

Between September 2014 and February 2015, we recruited children and youth aged 8 to 16 years with JIA cared for at the BC Children’s Hospital Pediatric Rheumatology clinical program to participate in the *LEAP-EI* study, a 6-month home- and group-based exercise intervention. The *LEAP-EI* study was a pilot intervention study as a component of a larger multisite (12 pediatric rheumatology centers across Canada), longitudinal observational cohort study of children and youth with JIA called *LEAP*; Linking Exercise, Physical Activity and Pathophysiology (*LEAP*) in Childhood Arthritis (*N* = 707). The *LEAP* study aims to investigate relationships between JIA, physical activity and bone and muscle development (www.leapjia.com). Inclusion criteria for the *LEAP-EI* were: diagnosis of JIA (either active or inactive disease), and willingness and physical ability to complete 3 days/week of prescribed exercise at home and participate in one group session/month, conducted by the exercise specialist at three community centres within the Greater Vancouver area. Exclusion criteria were: 1. receiving bisphosphonate treatment (past or present) for low bone mineral density, 2. high performance athlete (defined as participation in high performance sports, training or competition > 3 h/week), 3. currently participating in resistance training (defined as > 1 resistance training session/week for the past 4 months), and 4. pregnant or planning pregnancy. We invited 54 children and youth to participate in the *LEAP-EI*. Of these, 24 (44%) volunteered to participate. All parents provided informed consent and children and youth provided assent to participate in the *LEAP-EI*.

### Intervention

The 6-month intervention included home- and group-based exercise sessions, specifically designed for this project and adhering to current physical activity guidelines for muscle and bone strengthening exercises for children [7]. At home, we asked participants to complete jumping and hand grip exercises three times each week and resistance band exercises two times each week. Before beginning the exercise intervention, a kinesiologist visited each participant in their home to individually tailor the program to each participant’s current ability. Exercise intensity progressed across six, 4-week blocks from simple single joint exercises (e.g., bicep curl) to complex, multiple-joint exercises (e.g., lunges), with sessions ranging from an estimated 15–40 min. Participants were asked to attend one 60-min group exercise session each month at one of three locations across Vancouver BC’s Lower Mainland. In these sessions, the exercise specialist worked with each participant to progress their exercises for the next 4-week exercise block, and the participants also took part in group-based games. The exercise specialist contacted families by phone at regular intervals during the exercise intervention to provide support and answer family questions. At the end of each month she summarized her reflections about participants from the group sessions and calls. These notes were included in the qualitative analysis.

### Qualitative study design

We conducted separate pre- and post-intervention semi-structured interviews with children and their parents (and one grandmother at baseline, whom we describe as a ‘parent’ unless discussing their results separately) to explore their experiences and perspectives with the exercise intervention.

Dyads were invited to participate in the sub-study at the time of the child’s baseline bone and muscle measurements. We continued to recruit child and parent dyads were invited to participate in interviews until data saturation was reached [8], defined as the point when no new information emerged from the interviews. We determined data saturation based on our ongoing analysis of interview transcripts during the study by the lead author.

All children and parents who completed the exercise intervention were invited back for ‘follow-up’ interviews. In addition, children and parents who withdrew from *LEAP-EI* were invited back for ‘exit’ interviews.

#### Interviews

A trained interview moderator conducted the interviews. Interviews included general questions about leisure time activity as well as focused probe questions, with sample questions shown in Table 1. All interviews were conducted in a quiet room and were digitally recorded.

**Table 1:** Sample interview questions

1. What kinds of PAs/sports/exercises do you (or does your child) like to participate in? What do you (or they) like about these activities? 2. Do you have any concerns about (or your child) participating in sports or PA? If yes, what are they? If no, why not? 3. Tell me why you wanted to participate in this study? • Probe: Who made the final decision to participate? 4. Tell me what part(s) of the program you enjoyed? • Probe: exercises, group sessions, doing it at home 5. Tell me what part(s) of the program you disliked? • How did you overcome it during the program? • Describe what you would change to make it easier in the future?	

#### Reflective notes

The exercise specialist documented her own reflective notes regarding each participant’s adherence to the exercise intervention and monthly newsletter to the study team.

#### Adherence

To monitor adherence, we asked participants to complete a weekly exercise log, accessed either on paper or on line, on which they recorded number of exercise sessions completed (repetitions and sets), reasons for not completing exercises, pain experienced before and after exercising, perceived difficulty of the session, and any injuries. Adherence to exercise was defined as a percentage of the prescribed 6-month intervention completed (total reps completed/total reps prescribed). We excluded prescribed reps that children missed due to sickness, injury or being away at camp. Positive effects can be seen in physical activity programs where participants adhere to at least 60% of a program [9]. Therefore, for the purpose of this study, we classified adherence as *high* if participants completed ≥60% of the prescribed reps.

### Data analysis

Interview recordings were transcribed verbatim using a professional transcription service (Online and Ontime, Vancouver, Canada). We uploaded data into NVivo 10.0 (QSR International, Melbourne, Australia) for data management. We analyzed participant, parent and exercise specialist transcripts and reflective notes independently using a thematic 5-stage framework analysis: familiarization, identification, indexing, charting, and interpretation (Ritchie and Spencer, 1994). We identified two a priori themes; ‘Barriers’ and ‘Facilitators’ to exercise uptake and adherence. These themes created a structure for data interpretation. Finally, we integrated findings from thematic analyses of child, parent and exercise specialist interview transcripts, exercise logbooks and exercise specialist reflective notes to further advance our understanding.

## Results

### Characteristics of the study sample

#### Baseline

**Fig. 1:**
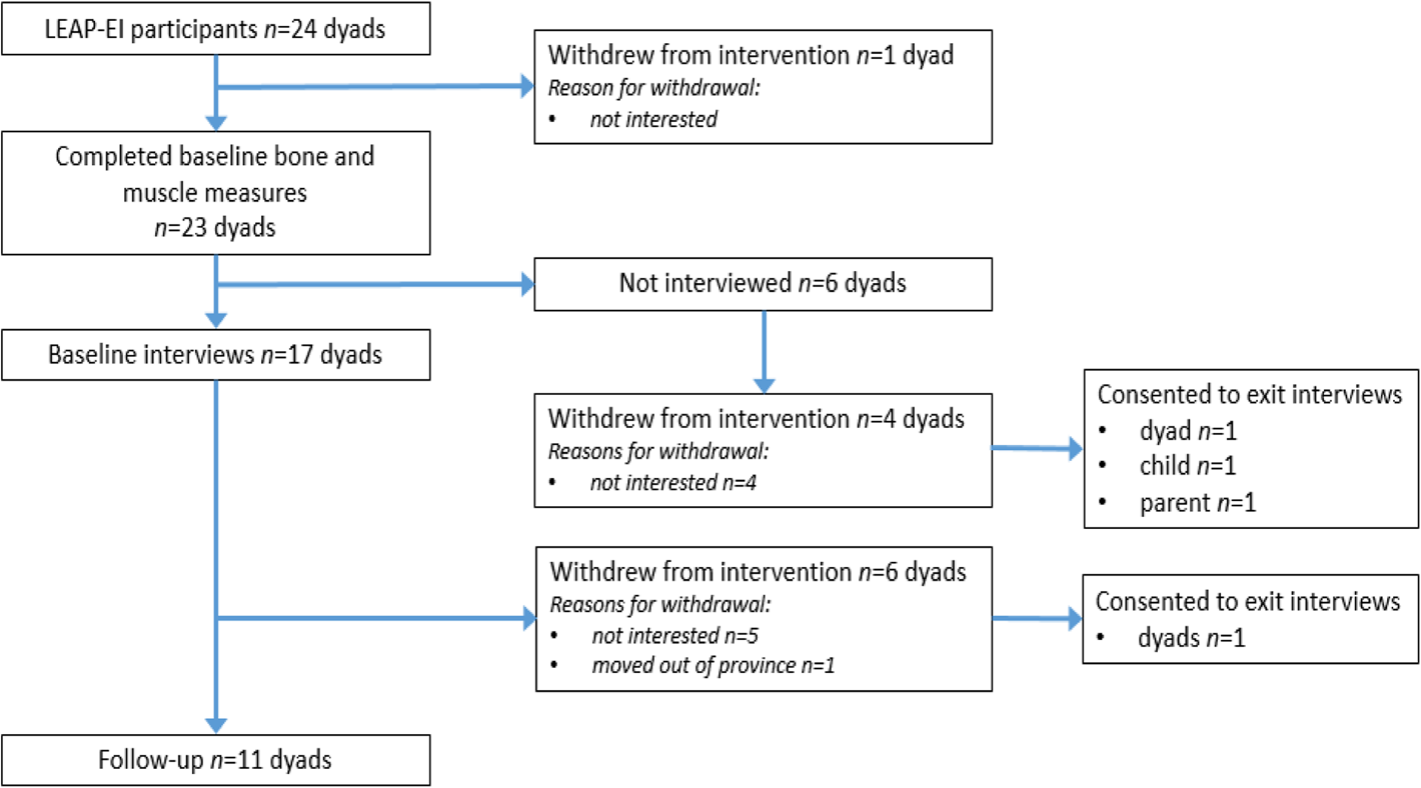
Flowchart that describes recruitment of participants into, and attrition of participants out of, the study

At baseline, we conducted 17 dyad interviews [17 child (8 girls, 9 boys) and 17 parent (13 mothers, 1 father, 1 grandmother, 2 mother/father pairs)] (Fig. 1). Child interviews required 36 min (range: 12–82 min) and parent interviews required 48 min (range: 21–84 min) to complete, on average.

#### 6-months

Of the 17 dyads interviewed at baseline, 11 completed their follow-up interviews and six withdrew from the intervention (5 for personal reasons, 1 moved). Informal discussions between study rheumatologists (KH and LT) and the five dyads who withdrew due to personal reasons identified a ‘lack of interest’ (low motivation, time constraints) as the primary reason for withdrawal. We attempted to conduct ‘exit’ interviews with these dyads; however, only one dyad consented. We also invited those dyads who withdrew from the exercise intervention (who did not participate in baseline interviews) to participate in exit interviews. One dyad, one child and one parent consented to an exit interview. Therefore, in total 18 complete dyads (child and parent) and 2 incomplete dyads participated in pre- and/or post-interviews for a total of 20 dyads.

We conducted follow-up interviews with 11 children (5 girls, 6 boys) and 11 parents (10 mothers, 1 mother/father pair) and exit interviews with 3 children (3 girls) and 3 parents (3 mothers) (See Fig. 1). Child interviews required 25 min (range: 10 to 55 min) and parent interviews required 34 min (range: 22 to 51 min) to complete, on average. We provide participant characteristics at baseline and 6-months in Table 2. In regards to adherence, participants completed a median of 46.9% (5.4, 66.7 IQR) of prescribed exercises, attended a median of 66.7% (16.7, 100 IQR) group sessions, and completed and returned 53.8% (19.2, 91.7 IQR) of logbooks (paper or online) [10].

**Table 2:** Demographic characteristics and exercise adherence (repetitions completed, attendance and logs received) for interviewed children and their parents

Dyad#	Sex	Pre/post child interview	Child baseline age (years)	Reps completed (%)	Group session attendance (range: 1–6 days)	Logs received (range: 0–26 weeks) (final week received)	Pre/post parent interview
1	M	Yes/Yes	11.8	1.9	2	5 (15)	Mom/Mom
2^a^	F	Yes/Yes (exit)	11.8	0.9	1	3 (3)	Mom/Mom (exit)
3	M	Yes/Yes	14.1	56.9	0	14 (26)	Mom/Mom
4^a^	M	Yes/No	11.1	1.8	1	2 (2)	Mom/no follow-up
5	F	Yes/Yes	10.2	46.9	6	23 (26)	Dad/Mom
6^a^	F	Yes/No	15.8	1.3	0	1 (3)	Mom/no follow-up
7^a^	F	Yes/No	12.3	1.7	1	4 (4)	Mom/no follow-up
8	F	Yes/Yes	14.6	58.3	5	19 (21)	Mom & Dad/Mom & Dad
9	M	Yes/Yes	14.6	15.8	5	9 (17)	Mom/Mom
10	M	Yes/Yes	10.5	47.5	4	17 (25)	Mom/Mom
11	F	Yes/Yes	10.6	82.3	5	24 (25)	Mom/Mom
12^a^	M	Yes/Yes	9.9	1.6	1	2 (2)	Mom/no follow-up
13	M	Yes/Yes	11.2	88.2	4	23 (26)	Grandma/Mom
14^a^	M	Yes/Yes	13.0	0	0	0 (0)	Mom/no follow-up
15	M	Yes/Yes	12.0	4.8	1	5 (6)	Mom/Mom
16	F	Yes/Yes	13.4	6.0	1	4 (4)	Mom/Mom
17	F	Yes/Yes	14.8	9.4	4	8 (14)	Mom & Dad/Mom
18^a^	F	No/Yes (exit)	16.4	2.5	0	2 (2)	no baseline/Mom (exit)
19^a^	F	No/No	10.0	0	0	0 (0)	no baseline/Mom (exit)
20^a^	F	No/Yes (exit)	12.8	0	0	0 (0)	none

^a^withdrew from the exercise intervention

### Thematic analysis

Three primary reasons children with JIA joined the study emerged from thematic analyses of pre-exercise intervention interview transcripts; 1) *potential health benefits*, 2) *selflessness,* and 3) *parental support*. Analysis of post-exercise intervention transcripts identified four main themes within a priori themes of intervention adherence. Facilitators were *parental support* and *enjoyment;* barriers were *time pressures* (subthemes: time required for exercise, scheduling, forgetting), and *physical ailments*. We describe the main themes and subthemes in Table 3.

**Table 3:** Summary of main themes from pre- and post-exercise intervention interviews: Facilitators (+) and barriers (−)

Themes and subthemes	+ or -	Description of themes and subthemes
Uptake		Factors that influenced exercise intervention initiation
Potential health benefits	+	Perceived health benefits of participating in the exercise intervention (i.e., increased fitness, disease management)
Selflessness	+	Study results may help other children with JIA
Parental support	+	Children’s perceived support from parents (i.e. encouragement, assistance) to participate in the exercise intervention
Adherence		Factors that influence continued engagement in the exercise intervention
Parental support	+	Parent support received by children (i.e. encouragement, assistance) during exercise intervention
Lack of enjoyment	–	Children’s dislike of the exercise intervention
Time pressures		Time pressures affected completion of exercise sessions;
*time required*	–	Total time required to complete exercise sessions
*scheduling*	–	Exercise session scheduling conflicts due to school, extracurricular activities and holidays/vacations
*forgetting*	–	Forgetting and missing exercise sessions
Physical ailments	–	Pain, injuries, illness that caused children to miss exercise sessions

### Factors that promoted uptake of the exercise intervention

#### Potential health benefits

Eleven children and 17 parents highlighted potential health benefits of exercise as a factor that influenced their decision to participate in the intervention. They believed that by participating, children would establish a regular exercise regimen and subsequently increase their level of physical activity and improve fitness, strength and overall quality of life. One boy described his desire to “*get better*”:

*“The thing is I want to get better. And if you guys can help me, you can. But that’s the reason [for participating]. I just want to get better, if you guys can help me.” (boy, 11 yrs).*

#### Selflessness

Nine parents and three children commented on how *“helping others”* motivated them to participate. They believed that study results may be used to better the lives of all children with JIA. A grandmother’s comment reflects this sentiment:

*“That’s why we’re doing it, the parents agree to do this, is because hopefully this research will help other kids as well.” (grandmother, grandson 11 yrs).*

#### Parental support

Ten children said parental support facilitated their participation. Despite not wanting to volunteer initially, some children participated because they knew their parents wanted them to. One girl explained:

*“Well, my mom definitely pushed that [exercise intervention], and then the girl [study recruiter] was there, I was just, like, you know, let’s try it. So I think, like, she [study recruiter] introduced it and she [mom] kind of pushed it a little bit.” (girl, 14 yrs).*

Children also noted that parental support (e.g., reminders, supervision, encouragement) was vital if they were to complete the prescribed exercises. One girl described her need for encouragement:

*“Well, I’ll definitely get my mom to push me too. I think I definitely need to be pushed to do things. It’s hard to remember to do them on your own.” (girl, 15 yrs).*

### Factors promoting adherence

#### Parental support

Parental support also emerged as a major facilitator of adherence. Parents were described themselves playing multiple roles, including motivator, assistant, coach/trainer, supervisor, liaison, timekeeper, record keeper and reward presenter. Six parents believed their involvement was key to their child completing the exercises. As one mother stated:

“*I think that it would not have happened if I wasn’t there pushing him and reminding him and prompting him. This would not have happened.” (mother, son 14 yrs).*

Two children with the highest adherence identified parent’s knowledge of exercise as a facilitator of program adherence. In both cases, the child’s parent had a university education in an exercise-related field (e.g., physical education, kinesiology). One boy described the value of having a knowledgeable parent to whom he could go for help:

*“Well, my dad’s really helped me out a lot though. Like, he is a P.E. teacher. Like, if I don’t understand the exercise or if I don’t know if I’m doing it right, then I ask him.” (boy, 11 yrs)*

The exercise specialist also stressed the importance of having a knowledgeable family member to whom children could go for advice:

*“Now those families that really succeeded, I think-- well, I say families because if the participant was working with one of their parents or another family member, they were doing a good job of getting the exercises done. I think that having at least one parent know what’s going on or how to do the exercises made it way better for the participant and their likeliness to actually be able to do it.” (exercise specialist).*

#### Lack of enjoyment

Lack of enjoyment emerged as a barrier to adherence. Ten children did not enjoy doing the exercises, particularly once the novelty wore off after the first few weeks. One girl who stopped logging exercises after week three and withdrew from the program soon after described her disinterest with the exercises:

*“She [the exercise specialist] came over one night and, like, walked me through everything. So, like, I knew how to do them. I just didn’t want to do them. Didn’t appeal to me.” (girl, 11 yrs).*

The exercise specialist also highlighted similar sentiments from the children in her reflective notes during the second month of the intervention:

“*It is starting to become apparent that some participants are bored or unmotivated to do their exercise. Some participants have said that it feels like doing homework, not fun, boring.*” *(exercise specialist).*

To counteract boredom, three children described listening to music or watching TV while doing the exercises. One girl stated:

*“I did the bands while I was watching TV, because it was, like, hard-- some of them were harder once I got into, like, seven exercises or something-- so, it was kind of boring without the TV.” (girl, 10 yrs).*

Exercising was low on some children’s list of competing priorities. Three children said they would rather do other things they enjoyed like playing, reading or hanging out with friends. One girl described activities she would rather be doing when at home:

*“When I’m at home. There’s so many things I would rather do, it’s like, I would rather finish this awesome book. I’d rather talk to my friend. I’d rather help this person out.” (girl, 14 yrs).*

Conversely, seven children reported liking some of the exercises:

*“I had one or two that I liked.” (girl, 10 yrs).*

Nine children and seven parents became frustrated with the resistance exercises and specifically, with tying the resistance tubing to stationary objects in their home. One mother explained her daughter’s frustration with the resistance tubing:

*“She complained about tying them. She found that a tricky one. She either tied them too tight and couldn’t get them off again, or she couldn’t get them to stay and when she started they would ping off whatever she’d tied them to.” (mother, daughter 14 yrs).*

Children and parents recommended ways to make the exercises more enjoyable including reducing the length and frequency of exercise to 3 days/week for 20–30 min/day and incorporating activities that the individual child enjoy. The intervention could also be modified by allowing children to supplement prescribed exercises with participation in other *“everyday”* sports/activities. One girl suggested:

*“Maybe not just [including] the same kind of resistance and the same kind of exercise. Like, different things, like, either swimming or running and not the same things. Biking, like, everyday activities more so.” (girl, 11 yrs).*

Although enjoyment was primarily described as a barrier to adherence there were aspects of the intervention that the children enjoyed. Nine children commented positively on the monthly group sessions; they enjoyed meeting and interacting with the other participants and looked forward to the friendly camaraderie during group activities.

*“I liked the group meet-ups, because you got to meet new people”. (girl, 10 yrs).*

Similar sentiments were highlighted by the exercise specialist.

However, not all children looked forward to the group sessions. One child who withdrew and one low-adhering child stated they felt anxious attending group sessions; they felt uncomfortable exercising with some of the other children whom they believed were more physically competent.

#### Time pressures

Time pressures emerged as barriers to adherence, including *time required* to complete exercises, *scheduling* time to do the exercises and *forgetting* to do the exercises.

##### Time required to do the exercises

Overall, most children (*n* = 8) and parents (*n* = 10) felt that the home exercise sessions were too long. Initially, resistance exercises took 10–15 min to complete. However, as the program progressed, children and parents reported taking 45 min to 2 h to complete an exercise session. Much of this time was spent attending to equipment difficulties such as tying/untying the resistance bands. One mother highlighted this issue:

*“And I’ll be honest, the resistance exercises were a lot harder for us to do, because it took a lot longer to do it. Whereas the other ones only took about five, ten minutes.” (mother, daughter 10 yrs).*

##### Scheduling

Five children and seven parents said it was difficult to find time during busy school and summer schedules to complete five exercise sessions per week and/or attend the monthly group session. One girl who stopped submitting logs after week two and subsequently withdrew explained that at the outset she did not realize the time commitment required and found it was impossible to manage due to her school work:

*“I realized I didn’t have enough time as I thought I would to do it. I didn’t do it as consistent as I should have. --I had a lot of [school work] ‘cause provincials-- I had to start studying way before because I’m not that school smart with some of my subjects.” (girl, 16 yrs).*

Holidays, family vacations, family visits and summer camps were other reasons children missed exercises and had difficulty returning to the prescribed routine. As highlighted by one mother:

*“It was much more doable during school days because then a routine was set. But during any holidays and weekends there is no routine. So the exercises kind of fall by the wayside.” (mother, son 14 yrs).*

##### Forgetting

Low-adhering dyads were more likely to forget to do the exercises. Three children (two low adhering) described forgetting to do the exercises, and six parents (five low adhering) said their child forgot to do their exercise and/or parents forgot to remind them. One parent expressed that remembering/reminding is a part of life when you have a child with JIA and that it can be difficult to remember everything:

*“I think for us it was hard to kind of remind her all the time. Remind her to take her medicine. Remind her to, you know, do her exercise program and she’s got all these other appointments in between, right?” (mother, daughter 13 yrs).*

#### Physical difficulties: Pain, injury and illness

Physical difficulties were the most commonly recorded barriers to exercise adherence in the children’s exercise logs. Children (*n* = 6), parents (*n* = 7) and the exercise specialist noted JIA-related pain (*“flaring” and “painful”* knees), non-exercise intervention related injuries (bruised hip, injured shoulder, car accident) and illness (flu, infection) as reasons for missing exercise sessions. In two girls, the prescribed jumping exercises caused significant knee pain. In consultation with study rheumatologists (KH, LT), the exercise specialist removed the jumping component from the girls’ prescribed exercises. One mother described an injury sustained during leisure time that caused her son to miss three weeks of exercises:

“*He fell playing paintball and had a massive, huge, out to there [shows with hands] contusion. So he was at the hospital, just to make sure that he didn’t wreck any internal organs.” (mother, son 14 yrs).*

## Discussion

Our results highlight that uptake of and adherence to a customized muscle-strengthening and bone-building exercise intervention for children and youth with JIA was influenced by a combination of personal, social and programmatic features. We move beyond studies that explored questions related to design of exercise interventions and their effectiveness and shift our focus to *why or why not* an intervention may or may not prove effective. Thus, we extend the existing literature to include multiple perspectives (children, parents, exercise specialist) related to barriers and facilitators to uptake of and adherence to a home-based exercise intervention.

### Uptake

Children and their parents highlighted potential health benefits of exercise and helping others as primary reasons for joining the study. This finding comes as no surprise, as children and their guardians most often identify health benefits and helping others as two key motivating factors for participation in pediatric research [11, 12]. Further, we would expect the potential for improved health to surface as a motivator for children with JIA and their families given the significant impact JIA has on children’s physical function and well-being. Children from the larger *LEAP* study also reported that physical benefits of physical activity facilitated physical activity participation [13].

We also identified parental support (desire for child’s participation and willingness to assist child during the intervention) as a major factor that influenced uptake in our cohort. This result is consistent with findings of others who identified parental support as a positive correlate of recreational and leisure physical activity in children and adolescents with disabilities, including JIA [14] and with healthy children [15]. It is therefore essential for future exercise studies in children with JIA to educate parents as to their critical role in facilitating their child’s participation and helping their child become more physically active.

### Adherence

Adherence was a significant challenge in our 6-month study. A combination of personal (lack of enjoyment, time pressures, physical ailments), social (parental support) and programmatic (lack of enjoyment, time pressures) factors influenced whether participants completed the prescribed exercises (or not). Our findings are consistent with results of previous studies of children with chronic conditions [6, 13, 16–18] whereby parental support facilitated participants’ physical activity participation/exercise adherence, especially among elementary school-aged children. In our cohort, parents adopted a number of supportive roles, including but not limited to, motivator, assistant and coach. In a study investigating parent involvement in a behavioural intervention that engaged their children, parents who were confident in delivering the intervention were found to be more involved [19].

Level of enjoyment is key to fostering recreational PA during childhood [20]. Children in our study emphasized that the exercise intervention was “*boring”* and *“hard”*. Two previous home-based exercise intervention studies involving children with JIA reported similar results [5, 6]. Three children in our study, and others [21, 22] created enjoyable distractions while exercising such as watching TV or listening to music. However, only one of these three children was considered high-adhering.

Time pressures also surfaced as a barrier to exercise adherence in this study, as in previous studies of children and adolescents with chronic conditions such as JIA [5, 6, 23]. Competing priorities such as vacation, after-school activities and homework were an issue for children. Time pressures may explain why children and parents identified “*forgetting*” as a barrier to adherence. Future exercise intervention studies with this population may benefit from incorporating time management strategies.

Finally, some participants in our study reported pain as a barrier to participation in the exercise program. This finding agrees with results from previous studies of children with JIA in which pain was identified as a barrier to participation in leisure-time physical activity [13, 24] and in exercise programs [5, 6]. In our program, the exercise specialist modified exercise prescription if a child reported pain (e.g. knee pain during jumping); this individualized monitoring and modifications minimized participant withdrawals due to increased pain.

### Implications for future research

We identified a number of important barriers and facilitators to participation in a home-based exercise program among children with JIA and their parents. Overall, our findings speak to the tension between efficacy and real-world trials. That is, although an exercise intervention must be designed to elicit a measurable system-level outcome response based on best evidence (in this case increased bone mass and strength and muscle strength) [10], it is imperative to also incorporate features that enhance participants’ enjoyment and willingness to participate. Our findings also speak to the importance of using qualitative methodologies within an implementation framework. In a knowledge to action cycle [25], findings can be continually fed back to those who designed and are delivering the intervention so that the intervention might be adapted to enhance adherence. Indeed, children and parents recommended important changes to the exercise intervention that they felt would make the program more enjoyable and less time consuming. Future studies should consider parallel mixed methods to obtain feedback from participants at more frequent intervals during the intervention.

### Limitations

Our study has a number of limitations. First, our sample was fairly small and we were unable to interview most dyads who withdrew from the exercise intervention (although we tried to do so). Thus, external validity of our findings may be limited. However, our findings revealed similar barriers to participation in home-based exercise as reported by others such as level of enjoyment [5, 6]. Second, three children were considered high adherers to exercise; we conducted interviews with two of these participants. Thus, there is a need to interview a larger sample of committed participants to better understand factors that encourage children and youth with JIA to engage with and adhere to an exercise intervention.

## Conclusions

Regular physical activity and exercise may help children with JIA manage symptoms, improve clinical outcomes and promote optimal growth and development. Despite the well-recognized benefits of physical activity, few children and youth with JIA currently engage in enough physical activity to achieve broad health benefits. Thus, researchers need to find a suitable compromise between what ‘works’ based on first principles of physiological response to exercise, and what is feasible to prescribe to children, youth and their parents based on what they find enjoyable, their many other interests, time commitments and a range of health limitations. Some answers reside within models of implementation that speak to the many factors beyond the intervention itself that need be considered for any intervention to be effective [26, 27].

## Abbreviations

JIA: Juvenile idiopathic arthritis
LEAP: Linking Exercise, Activity and Pathophysiology
LEAP-EI: Linking Exercise, Activity and Pathophysiology in juvenile idiopathic arthritis: Exercise Intervention

## Footnotes

1 Caspersen et al. define physical activity as “any bodily movement produced by skeletal muscles that results in energy expenditure” (p.126) whereas exercise is defined as a “subcategory of physical activity that is planned, structured, repetitive and purposive” (p.128).
